# EEG Signatures of Melancholia: An Update

**DOI:** 10.3390/neurosci7030074

**Published:** 2026-06-21

**Authors:** Christopher F. Sharpley, Vicki Bitsika, Christopher B. Watson

**Affiliations:** Brain-Behaviour Research Group, University of New England, Armidale, NSW 2351, Australia; csharpl3@une.edu.au (C.F.S.);

**Keywords:** depression, melancholia, EEG, electrophysiology

## Abstract

Melancholia remains a severe and complex form of depression. One possible avenue to a better understanding of melancholia and potentially improved methods of treating it, is via examination of the profiles of brain electrical activity of patients suffering from melancholia. However, apart from work using fMRI, relatively little is known about the electrophysiological basis of melancholia despite the potential for this to inform targeted effective treatments such as Transcranial Magnetic Stimulation. To better understand the state of research regarding EEG variables and melancholia, a systematic review was undertaken. Results indicated that there was a large degree of complexity in the association between melancholia and various EEG parameters, and that many specific aspects of brain electrical activity remain under-studied. Suggestions are made for future research.

## 1. Introduction

### 1.1. Depression

As well as causing significant distress to its sufferers, Major Depressive Disorder (MDD) [1] contributes substantially to the global disease burden [2] and is often comorbid with other major illnesses and disorders [3,4,5]. MDD is also linked to accompanying decreases in cognitive abilities [6] social relationships [7] functional capacity [8] and quality of life (QOL) [9,10]. Although it is defined as a unitary disorder, MDD has highly heterogeneous symptomatology [11,12,13,14], resulting in attempts to define subtypes of MDD that may enhance the accuracy of diagnosis and treatment. One of those subtypes is melancholia.

### 1.2. Melancholia

Melancholia has a long history, based in the ancient Greek word μελαγχολία, meaning black bile, as a signifier of the severity of the effect of the distressed mental state upon physical function. Consequently, the biological nature of melancholia has been emphasized throughout history, particularly in the dichotomy between depression with a biological cause (referred to as ‘endogeneous depression’) and depression as a reaction to environmental stress (‘exogenous depression’) during the twentieth century. Although the endogenous vs. exogenous distinction has now become less common in diagnostic practice, melancholia remains characterized by anhedonia, psychomotor disturbances, and neurovegetative symptoms [15,16,17], arguing that it is a distinct form of depression from MDD. However, its detailed symptomatology has been variously defined during the last 50 years [18] and the subject of some argument [19]. As a result, reviews of melancholia have often commented on the challenge of drawing firm conclusions about correlates of melancholia based upon studies that applied different measures of melancholia, or even included different forms of depression that were assumed to overlap with melancholia.

### 1.3. Defining Melancholia

Several attempts have been made to identify and define melancholia as different to MDD via biological factors such as hypothalamus–pituitary–adrenal dysfunction and a poor response to placebo medication but having a higher likelihood of responding to antidepressant medication and electroconvulsive treatments than to psychotherapy [20]. More recently, Parker and colleagues have reported on a valid and reliable definition and measure of melancholia as distinct to MDD-- the Sydney Melancholia Prototype Index (SMPI) [17,18,21,22]. The SMPI relies on symptoms such as low energy, severe anhedonia, and apathy, all of which are worse in the morning. Other symptoms include psychomotor retardation, difficulty concentrating, weight loss, and more severe depressed mood than would usually be present in MDD, enabling the SMPI to be used to differentiate melancholic depression from non-melancholic depression [17,23,24]. However, these symptoms do not include neural phenomena, which might be assumed to be the core feature of any mental state. As such, some attention has been given to identifying neurological factors that are correlated with melancholia.

For example, three relatively recent reviews focused on the neurological correlates of ‘melancholia’ [22,25,26], although only Esposito and Buoli [26] (p. 55) exclusively reviewed studies that described ‘melancholia’ specifically, while Spoelma et al. [22] and Bruun et al. [25] covered a wide range of possible biological correlates of a range of forms of depression, including ‘endogenous depression’ which did not include the SMPI range of symptoms, and thus may not have been melancholia as defined by Parker et al. [21].

### 1.4. EEG and Melancholia

Both Esposito and Buoli [26] and Spoelma et al. [22] gave minimal coverage of actual brain variables (principally as measured via fMRI), but Brunn et al. [25] focused exclusively on the electrical activity of the brain as measured by electroencephalographic (EEG) methods. Although fMRI provides excellent spatial identification of oxygenated blood flow, EEG is more accurate temporally and in reporting electrical activity in the brain, leading to the combination of both methods (reviewed by [27]), although this procedure is not without major challenges, particularly since each method can interfere with the other [28]. Additionally, the inability of fMRI data to describe underlying electrophysiological activity in the brain [29,30] produces an argument for the use of EEG when attempting to define the brain’s electrophysiological correlates of melancholia. Although some attention has been given to the changes in EEG signals during various tasks, resting EEG data have been demonstrated to provide the most reliable indicator of the participant’s usual state when investigating psychiatric disorders such as melancholia [31,32,33], and therefore this review used the presence of resting EEG data as an inclusion criterion.

### 1.5. Study Aims

This review broke new ground in the attempt to understand one of the most challenging aspects of depression—melancholia. In a novel approach to this challenge, it focused upon resting EEG data as a correlate of melancholia in an attempt to better understand the electrophysiological substratum of melancholia, and to update the review by Esposito and Buoli [26]. As well as potentially informing understanding and treatment of melancholia in ways that have not been previously attempted, the review aimed to provide a reliable indicator of the electrical behaviour of the brain in human participants who met the criteria for melancholia defined in the SMPI (or a reasonably similar criteria). The findings from such a review could contribute towards more effective use of one of the most successful therapies for treatment-resistant depression via Transcranial Magnetic Stimulation (TMS) [34,35] by identifying specific brain regions for stimulation because of their association with melancholia. TMS has been shown to reduce symptom severity in melancholia patients [36,37], thereby establishing a plausible potential link between the results of this review and treatment opportunities.

## 2. Materials and Methods

### 2.1. Search Strategy

**Figure 1:**
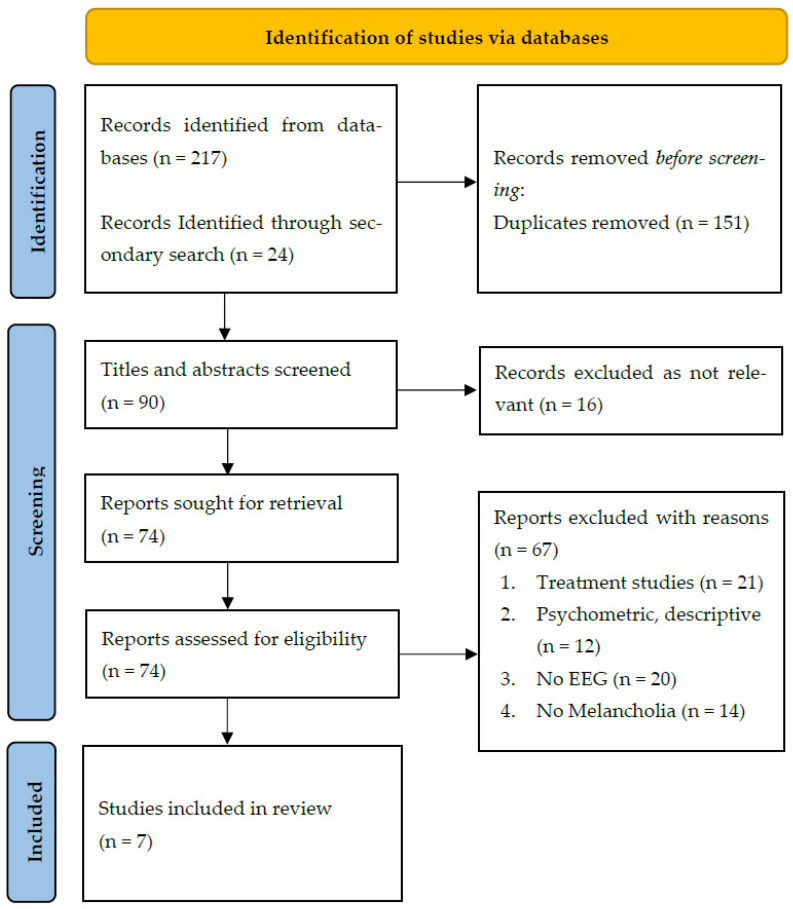
PRISMA flowchart of search and selection process for studies examining EEG correlates of melancholia.

A search was conducted in PubMed, Embase, PsychInfo, and Scopus using broad terms including ‘EEG’, ‘Melancholia’ and ‘depression’ (the full search query for each database is available in Supplementary Material S1). This search was conducted for the period 1 January 2019 to 28 February 2026. Esposito and Buoli [26] finished their search in September 2019, and so the current search commenced in January 2019 to reduce the likelihood of missing some relevant papers. Studies identified by this procedure were added to those reviewed in Bruun et al. [25], Esposito and Buoli [26], and Spoelma et al. [22] that used EEG. This review was conducted in accordance with the Preferred Reporting Items for Systematic Reviews and Meta-Analyses (PRISMA 2020) guidelines. Figure 1 portrays the study selection process followed and the completed PRISMA checklist is available in Supplementary Material S2. The search protocol was not registered with PROSPERO because a review of diagnostic/treatment biomarkers does not meet the eligibility criteria of having direct clinical significance. Furthermore, only intervention-focused reviews are permitted to be registered at this time.

### 2.2. Inclusion and Exclusion Criteria

Apart from reporting on resting EEG and applying some form of melancholia assessment that approximated the SMPI, the other inclusion criteria was that only English language studies using adult humans were reviewed. Studies that reported on ‘endogenous depression’ were not included after scrutiny of the diagnostic criteria they applied, which were in no cases congruent with the SMPI list of melancholia symptoms. Studies were excluded during full-text screening if they were examining a treatment approach, if they were only psychometric or descriptive, if they did not involve EEG, or if they did not assess melancholia. Two independent reviewers (CFS, VB) scrutinized each study, and any disagreements were discussed and resolved.

### 2.3. Quality and Risk of Bias Assessment

The quality of studies and risk of bias was evaluated using the Newcastle–Ottawa Quality Assessment Scale for Case–Control Studies to measure the adequacy of case definitions, representativeness of cases, selection and definition of controls, how well the study controlled for confounders, the method used to ascertain exposure, and whether the same method was used for both cases and controls. Studies were rated on a scale of one to nine stars, with ‘high-quality studies’ with a low risk of bias defined as greater than seven stars, ‘moderate’ as five to seven stars, and ‘low’ as less than five stars. The quality assessment was undertaken by two independent reviewers (CFS, CBW; see Supplementary Material S3).

### 2.4. Data Extraction and Analysis

Descriptive information was extracted separately by two reviewers (CFS, CBW) and was entered into a summary table. This information included the name of author/s and year of publication, melancholic, non-melancholic and control sample sizes, number of males and females, age (mean), diagnostic protocol used for melancholia, the electroencephalographic variable assessed, effect outcomes and effect size estimates. Where effect size values were not provided by the study, calculations were performed autonomously by each reviewer (where sufficient data were available) to ensure accuracy.

A meta-analysis was not performed due to the small number of studies identified. Furthermore, the substantial variability in how melancholia was measured and the range of EEG characteristics assessed suggests that a pooled estimate would not represent a clinically meaningful common effect.

## 3. Results

### 3.1. Studies Identified

The primary database search identified 217 studies, and a secondary manual search located a further 24 research articles. The comparison of titles and author names resulted in 151 duplicates being removed and the abstracts of the remaining 90 studies were then evaluated for relevance. Overall, 74 articles were downloaded in full text for close assessment and seven were ultimately selected for review. The search and refinement process described above produced the studies summarized in Table 1.

### 3.2. Diagnostic Protocols Used in Research Studies

Although the major details of the seven studies that met the inclusion criteria are shown in Table 1, some further comments are necessary regarding the methods used by those studies to measure melancholia. The two studies by Pizzagalli and colleagues [38,39] and the study reported by Shankman et al. [40] applied the DSM-IV diagnostic criteria for melancholic features; Quinn et al. [41] used the MINI International Neuropsychological Interview (an alternative to the DSM-IV Melancholic Features subtype of Major Depression) and the CORE Assessment of Psychomotor Change, derived by Parker et al. [45]; and Sharpley [42,43,44] applied the diagnostic criteria from the SMPI.

Table 2, column 1 presents the DSM-IV diagnostic features for Depressive Disorder with Melancholic Features, and column 2 shows the CORE items that Quinn et al. [41] used to confirm initial diagnosis of melancholia obtained via the MINI (which is an alternative for the DSM-IV Melancholic Features scale). In column 3, the SMPI criteria described by Parker et al. [17] are shown for comparison.

These three sets of diagnostic criteria emerged from different processes. The DSM-based diagnostic criteria were developed to identify a subtype of MDD which demonstrated several extra features associated with melancholia. Thus, the aim of that process was to better understand how MDD might vary while remaining MDD. The MINI is used to apply the DSM-IV diagnostic criteria for MDD and may also be used to detect Major Depression with Melancholic Features (see column 1, Table 2). By contrast the CORE system is composed of a series of mental signs that Parker and colleagues developed to distinguish the clinical features of depression types that responded better to physical treatment, and which was “varyingly termed endogenous, melancholic, psychotic, severe, vital, autonomous and endogenomorphic depression” [45] (p. 55). Of note, Parker et al. [45] included psychotic and endogenous depression as subtypes of melancholia, and focused on clinical ‘signs’ (objective evidence or physical manifestation, such as immobile face, slumped posture, slowed movements, and reduced speech, among others: see Table 1, Parker et al. [45] for a full list) and ‘symptoms’ (generally reported by the patients and not always observable, such as severely depressed mood, indecisiveness, appetite loss, slowed thoughts, anhedonia, and others: see Table 4, Parker et al., [45]) as different classes of clinical evidence. Some 27 years later, Parker et al. [18] summarized the research literature on the use of the CORE to define melancholic patients, concluding that it could “be used to enrich the standard clinical interview of depressed patients when a probability estimate of melancholia is sought, either for research or clinical purposes” (p. 133). The SMPI has undergone repeated psychometric evaluation and refining to produce what Parker et al. [17] referred to as a measure allowing “firm clarification as to whether melancholic and non-melancholic depression differ” (p. 73). The SMPI has robust psychometric evidence to support its use in clinical and research settings.

### 3.3. Study Findings

Pizzagalli et al.’s [38,39] studies followed two different research aims, although based on the same sample of 20 melancholia, 18 non-melancholia, and 18 non-depressed unmedicated participant volunteers recruited from the local community in Madison, Wisconsin via media advertising. The mean ages of all three subgroups were not significantly different, nor were the subgroups significantly different in terms of sex or sociodemographic factors. Diagnosis of melancholia, non-melancholia, or no depression were based upon multiple Structured Clinical Interview for Depression (SCID) interviews [46] adjusted for DSM-IV to focus upon MDD Melancholic Features. The first of Pizzagalli et al.’s studies reported on frontal electrical activity and found no significant differences according to melancholia status. The second of Pizzagalli et al.’s studies tested the hypothesis that melancholia would be associated with reduced electrical activity in the subgenual prefrontal cortex (PFC) and found that melancholic participants showed more delta wave activity in the subgenual PFC than either the non-melancholic depressed or the non-depressed cohorts. This finding confirmed the observation made by Austin et al. [47] that the areas of the PFC that are dopamine-rich are the primary sites of dysfunction in melancholia, although Pizzagalli et al. [38] noted that their findings questioned the homogeneity of melancholia.

Shankman et al. [40] also recruited volunteer community participants via media advertising, but in the New York area. Diagnosis of melancholia was based on DSM-IV criteria plus selected items from the Hamilton Rating Scale for Depression (HRSD) including the symptoms of middle insomnia, late insomnia, work and activities, retardation, agitation, loss of weight, diurnal variation and hopelessness. Shankman and colleagues compared frontal and posterior asymmetry between melancholics and non-melancholics (there was no difference in the proportion taking psychotropic medication) and non-depressed participants, but also included anticipatory and consummatory anhedonia as a variable by placing participants in a slot wheel spin reward situation which participants either won or lost. *Post hoc* analyses revealed that there were no significant differences in frontal alpha asymmetry before receiving the reward, but after having gained their rewards melancholia participants showed greater left posterior activity while non-melancholia participants showed greater right posterior activity. One major limitation of these findings for the present review is that the pre-reward condition may not have accurately represented a true rest condition because participants were aware of the impending experiment. While this may not be a serious issue because almost all research participants are in a state of some anticipation regarding the experimental protocols they may encounter, there is some doubt as to the complete validity of the findings *vis-à-vis the* presence of asymmetry as a correlate of melancholic depression.

Quinn et al. [41] accessed data from 47 melancholia patients, 50 non-melancholia depressed patients (all unmedicated), and 120 healthy control participants from the Brain Resource International Database (USA, UK, Holland, South Africa, Israel and Australia). The MINI was used to identify depressed and non-depressed participants, and also to delineate melancholia from non-melancholia depressed participants. This division was confirmed by application of the CORE criteria. EEG data were collected during a 2 min rest period and analyzed for alpha asymmetry differences in frontal, temporal, and posterior regions across the three participant subgroups. There were no significant differences in any of these asymmetry indices between the melancholia and control subgroups. Quinn et al. [41] commented that the previously reported association between alpha asymmetry and depression may have been due to the presence of differing levels of anxiety across participant subgroups.

Sharpley and colleagues [42,43,44] performed a series of sequential studies on different aspects of EEG data collected from the same group of 100 unmedicated community volunteers in the New England region of New South Wales, Australia. Participants were classified as depressed (*n* = 33) versus non-depressed (*n* = 67) via their total raw score on the Zung Self-rating Depression scale (SDS) following Zung’s criterion of a raw score > 39 as the cutoff for clinically significant depression [48]. An eight-item scale using SDS items plus several items derived from the SMPI was used as diagnostic criteria for melancholia (anhedonia, low energy, loss of interest, impaired concentration (two items), thoughts of death or suicide (two items), and not being able to be cheered up even when good things happened) to identify melancholia versus non-melancholia participants based on the SMPI symptomatology.

Because of the inconsistent results from previous studies on alpha asymmetry and melancholia, the first of these three studies [42] focused on that variable. During the course of their data analyses, this team became aware of a dichotomy in the results: three frontal EEG site pairs (F4-F3, FC4-FC3, FT8-FT7) showed significant inverse correlations with specific items from the melancholia scale, and one posterior EEG site pair (PO2–PO1) was significantly directly correlated with two different items from the melancholia scale. When the eight melancholia scale items were subject to factor analysis, the resulting solution produced two factors, which aligned with the different direction and site EEG asymmetry results. Those two factors were: *Fatigue-withdrawal* (significantly inversely correlated with frontal alpha asymmetry) and *Social reasoning*, *misinterpretation* (significantly directly correlated with posterior alpha asymmetry). These findings may reveal possible sources of the previous inconsistent findings regarding alpha asymmetry and melancholia by suggesting that the eight-item melancholia scale based on the SMPI was, in fact, a composition of two subscales that measured different aspects of melancholia.

The second study reported by Sharpley et al. [43] aimed to investigate the association between fast versus slow wave resting EEG data and each of the eight melancholia items on the melancholia scale that was derived from the SMPI. The particular EEG sites observed were the occipital–parietal PO1 and PO2, and the frequency ranges examined were theta (4 Hz to 8 Hz), alpha (8 Hz to 13 Hz), and beta (13 Hz to 30 Hz) during 3 min of rest. The major dependent variables were theta:beta ratio and alpha:beta ratio, enabling a ‘slow:fast’ comparison of electrical activity to be made. Results indicated that the slow:fast comparisons were directly correlated with melancholia item “Others would be better off if I was dead” at PO1 for depressed participants but not for non-depressed participants. That is, depressed participants had more slow wave activity in the PO1 region than non-depressed participants but only for the melancholia symptom that indicated feelings of worthlessness and thoughts of death or suicide. These results were replicated but with a weaker correlation coefficient at PO2, suggestive of the presence of reduced beta wave activity in the parietal–occipital region for depressed participants. Because this region is associated with the cognitive activities of coding episodic memory and evaluating sensory information in regard to one’s goals, it may be that these results reflect the relative disengagement from environmental stimuli that has been suggested to underlie MDD [49,50] and which may represent a major aspect of melancholia.

Sharpley et al.’s third study examined the relationship between gamma waves and melancholia symptoms. By subdividing the gamma frequency range (30 Hz to 130 Hz) into 20 Hz sub-bands, it was apparent that different melancholia symptoms were associated with different gamma sub-bands at a range of EEG sites. The overall finding was that these correlations were direct, indicating that, as gamma power increased within various sub-bands, so did the severity of the melancholia symptoms that were specifically associated with those gamma sub-bands.

**Figure 2:**
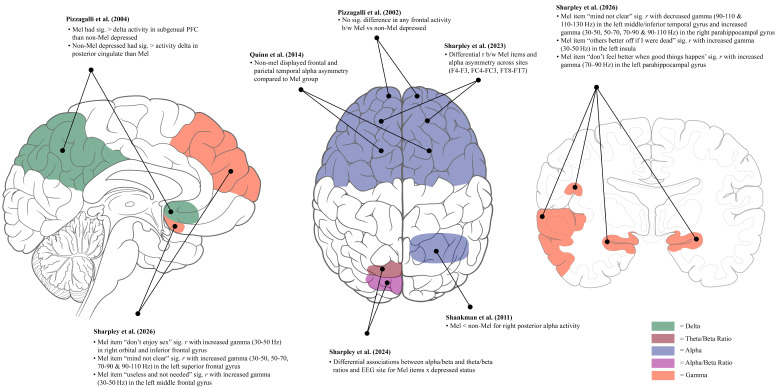
Brain regions and research findings, melancholia total and item scores.

Figure 2 provides a visual summary of the findings reported in Table 1, enabling clearer understanding of which brain regions have been previously investigated. It is apparent that previous research found associations between EEG-based data and melancholia in only restricted brain regions. When the total score on a melancholia inventory was used as an indicator of melancholia, the presence of melancholia was not accompanied by significant differences in frontal activity for depressed participants; similarly, when a clinical diagnosis was used, there was a contradiction in findings reported by Pizzagalli and colleagues between their 2002 and 2004 studies. As well as representing the relative lack of systematic investigation of the association between melancholia and electrical activity across all regions of the brain, Figure 2 also indicates that melancholia’s association with the brain’s electrical activity may not be most accurately understood as a unitary or homogenous link. Instead, there is some evidence of different brain region activity being correlated with different symptoms of melancholia. Added to that heterogeneity in the association between the brain’s regional electrical activity and melancholia symptoms is the clear finding that there has been no consistent investigation of the association between different brain regions and melancholia across the whole range of brain wave bands (i.e., theta, alpha, beta, gamma). On these bases, the detailed and specific representation of how the electrical activity of the human brain interacts with the various symptoms of melancholia remains largely open, with only a few initial findings reported so far.

## 4. Discussion

### 4.1. Major Findings

Overall, these seven studies emphasize the complexity and the limitations in the research findings on the association between EEG activity and melancholia and its symptoms. Although there is some initial evidence of differences in brain region asymmetry between melancholics and non-melancholic depressed patients, there is a good deal of inconsistency in that result across the studies that focused on asymmetry [40,41,42], so that it is premature to definitely conclude that asymmetry metrics might be applied as a biomarker of melancholia. The papers by Pizzagalli et al. [39] and Sharpley et al. [43] produced results that suggest that delta power and the ratio of theta to beta, and alpha to beta may hold some promise in identifying melancholia, but those results need further confirmation before they may be accepted. The papers by Sharpley and colleagues [42,43,44] provide initial impetus to consider melancholia from the perspective of the individual symptoms comprising it rather than using a total score from an instrument such as the SMPI.

The recognition that melancholia (like MDD) is heterogenous in terms of the associations between individual symptoms and EEG data has not been previously discussed, although the heterogeneity of MDD symptoms (which are similar to those used in the SMPI) is accepted (e.g., [51,52,53]). That heterogeneity of MDD has been described as “hampering scientific research and limiting effectiveness of therapeutic interventions” [52] (p. 559). Similarly, so may it be said that the heterogeneity of melancholia is a major challenge to the development of a more detailed understanding of this complex phenomenon and its association with brain activity, and its effective treatment.

As mentioned earlier in this paper, there have been several reviews of the large set of possible biomarkers of some definitions of melancholia, but relatively little attention given to the electrophysiological correlates of melancholia via EEG. What has been reported from that limited field of studies is largely based upon the definition of melancholia as a homogeneous construct. The present review argues that this is a potentially frustrating search because of the different associations between melancholia symptoms and different forms of electrical activity across multiple regions of the brain.

By reference to Table 1 and Figure 2, it is apparent that selected regions of the brain have been targeted for EEG research on the correlates of melancholia. That selectivity may be based upon hypotheses drawn from previous research (e.g., frontal asymmetry and MDD) but carries with it the limitation that the entire brain has not been systematically studied for the range of associations between melancholia symptoms and EEG data. This is clearly a major and preferred target for future research to advance diagnosis and treatment of melancholia.

Overall, the present state of knowledge regarding brain electrical activity and melancholia is in its infancy. The few conclusive findings indicate that there is variability across brain regions and frequency ranges of the common ‘bands’ that are used to categorize brain activity. This finding reveals a complexity that is yet to be defined in detail but does support the further search for EEG-based correlates of melancholia. That search should not be limited to simple EEG signal power or ratio data but needs to also include some of the more recent EEG variables such as cross-frequency coupling, machine learning, and neuromodulation effects.

### 4.2. Implications for Research and Practice

The range of different findings across these studies challenges the validity of using a single cumulative measure of melancholia symptoms when trying to identify the neurophysiological bases of this particular form of depression. These preliminary data suggest that melancholia’s different symptoms may each possess their own EEG signature, but that is still yet to be demonstrated conclusively. In this way, melancholia may reflect the heterogeneity of MDD itself [54,55]. As such, emphasis in clinical assessment and treatment should follow a recommended ‘personalized medicine’ model [56,57]. That is, one in which each patient is considered from the perspective of their symptom profile rather than a total score on a self-report inventory or a classification arising from a clinical interview based on dichotomous symptom presence. However, at this stage, reliance on particular EEG signatures of melancholia symptoms is premature.

## 5. Limitations

Relatedly, it must be acknowledged that this review purposely focused on EEG data because relatively little had been previously reported regarding this index of brain electrical activity. It must also be acknowledged that a great deal of research has been conducted using other potential biomarkers of melancholia, such as fMRI, inflammation, hormonal indices, and other physiological variables. These are all valuable in the search for neurobiological correlates of melancholia, and no suggestion is made here that they do not contribute in meaningful ways to understanding this particular from of depression. However, as argued in the early sections of this review, they do not report on the actual electrical activity in the brain, which may be argued as a more relevant indicator of how that organ is linked to the psychiatric disorder of melancholia.

A major current limitation on any review of the resting state EEG–melancholia literature is the relatively small number of relevant studies available to review at this time, and the use of the same dataset across some studies. This is, in itself, a significant finding because it argues for a concentrated effort to investigate this important correlate of melancholia. Although the latter is not a major argument against accepting the findings from these studies because the study foci were varied, there is a question of generalizability across cultures and populations that have not been recruited into these studies. A such, these factors argue against any firm conclusions being drawn about the nature of the relationships between EEG data and melancholia yet. As noted in the Introduction, a major recent review of the EEG correlates of melancholia [25] identified only five resting state studies, all of which are included here except one which recruited only participants with affective disorders [58], and another which included dementia patients [59]. Other aspects of EEG that were reviewed by Bruun et al. [25] included 10 sleep studies and nine event-related potential studies. Brunn et al. [25] commented that, within each of these three sets of different methodologies, studies “were characterized by marked variability on almost all levels, preventing pooling of data” (p. 1). Although, as argued in the Introduction, resting state EEG provides the most reliable indicator for psychiatric disorder melancholia [31,32,33], other EEG data might repay further investigation for their association with melancholia. A further caveat regarding the use of resting state EEG is the limitation of EEG in terms of spatial resolution, although this is largely compensated for by use of data analytic software such as eLoreta [60].

In terms of measurement of melancholia, Table 2 presented the different symptomatology used across three major instruments in the studies reviewed herein. While it is clear that there is some overlap across these three instruments (e.g., anhedonia, unable to be cheered up), there is also a considerable degree of difference between the individual symptoms displayed for each measure. Unfortunately, because studies that used these different measures also focused on different aspects of resting EEG, no direct comparison or evaluation of the effect of different measures could be ascertained. This remains a task for future research. Similarly, anxiety may BE a potential confound for EEG measurement protocols and must be included as possibly accounting for the lack of clear-cut findings in the seven studies reviewed here.

Other avenues for future research include the possible confounding effects of antidepressant medication, benzodiazepines, mood stabilizers, and other psychotropic treatments on EEG measures. Among the seven studies reviewed herein, all used unmedicated participants except for Quinn et al. [41] where there were equal proportions of medicated participants among the depressed subgroups, making comparison of medicated vs. non-medicated participants impossible here.

## 6. Conclusions

By focussing on how the brain’s electrical activity interacts with the various symptoms of melancholia, this review aimed at the core of understanding melancholia as a neurophysiological phenomenon *per se*. However, although the prevalence and severity of melancholia argue for its systematic investigation, the present review indicates that more work is needed before firm conclusions can be drawn. It is clear that, at present, this investigation is in its early stages and has been largely undertaken in a piecemeal fashion. Although some valuable findings resulted from the seven studies reviewed here, the disparate research foci of those seven studies reflect a lack of planned concentration that followed a systematic investigative plan. The most important implications from this review are that, apart from seven studies with distinct and unrelated research aims, only a relatively sparse model of brain-related melancholia phenomena is currently available. What is now required for this field to advance so that it can make a realistic and valuable contribution to the understanding and treatment of melancholia is systematic focused EEG-based research using a standardized valid scale for measuring melancholia, and to undertake that search with reference to the range of EEG data and the breadth of melancholia symptomatology.

## Figures and Tables

**Table 1:** Summary of resting state EEG studies on melancholia.

Study	Melancholic Patients (*n* Female) [Age (yr)]	Non-Melancholic Patients (*n* Female) [Age (yr)]	Controls (*n* Female) [Age (yr)]	Diagnostic Protocol	EEG Variable	Results	Effect Size	Conclusion
Pizzagalli et al. [38]	20 (13) [*M* = 36.5, SD = 12.9]	18 (10) [*M* = 33.1, SD = 8.8]	18 (10) [*M* = 38.6, SD = 13.6]	DSM-IV for Melancholic Features	Frontal activity	Ns difference in frontal activity b/w Mel ^5^ vs. non-Mel depressed (*p* > 0.45)	Insufficient data	Melancholia not linked with increased frontal activity
Pizzagalli et al. [39]	As in Pizzagalli et al. [38]				Delta activity	Mel had sig. > delta activity in subgenual PFC than non-Mel depressed Non-Mel depressed had sig. > delta in posterior cingulate than Mel	Cohen’s *d* = 0.87 Cohen’s *d* = −0.98	More delta activity in frontal region for Mel Ss Less delta activity in posterior region for Mel than non-Mel
Shankman et al. [40]	17 [*M* = 35.5]	48 [*M* = 33.2]	34 [*M* = 33.8]	DSM-IV for Melancholic Features ^1^	Frontal and posterior alpha asymmetry	No sig. diffs b/w Mel and non-Mel for frontal asymmetry Mel < non-Mel for right posterior activity	*F* [2, 96] = 1.14, *ns* *F* [1, 63] = 6.83, *p* < *0*.01, η^2^_p_ = 0.07	Alpha asymmetry sig. greater in Mel than non-Mel at posterior but not frontal region
Quinn et al. [41]	57	60	120	MINI ^2^ CORE ^3^	Frontal, parietal-temporal, posterior asymmetry during eyes closed, 2 min rest	Non-Mel sig. greater asymmetry at frontal and posterior than either Mel or Control. No sig diffs b/w Mel and Control for asymmetry at any site	Ear reference Cohen’s *d* = 0.34 Average reference Cohen’s *d* = 0.33	Mel showed ns differences in asymmetry than Control across brain sites
Sharpley et al. [42]	33		67	SMPI ^4^	Frontal, parietal–occipital alpha asymmetry during 3 min eyes closed rest	Total Mel score (8 items) created 2 factors (Fatigue-withdrawal; Social reasoning, misinterpretation). Differential *r* b/w Mel items and alpha asymmetry across sites	FP2–FP1 (*r* = −0.388, *p* = 0.026) F4–F3 (*r* = −0.413, *p* = 0.017)	Mel is not unitary. Mel symptoms require assessment rather than total score based on alpha asymmetry
Sharpley et al. [43]	As for Sharpley et al. [42]				Ratio of alpha/beta and theta/beta at PO1, PO2	Differential associations between ratios and EEG site for Mel items x depressed status	Theta/Beta D PO1 rho = 0.54 Alpha/Beta D PO1 rho = 0.43	Mel is not unitary. Mel symptoms require assessment rather than total score based on ratios of alpha/beta and theta/beta
Sharpley et al. [44]	As for Sharpley et al. [42]				Correlation b/w gamma sub-bands and Mel items	Differential associations b/w gamma sub-bands and Mel at specific sites	Multiple sites and frequencies *r* > 0.296	Complex association b/w gamma frequency bands and Mel symptoms

^1^ See Table 3, ref. [41]; ^2^ MINI = Mini International Neuropsychological Interview (used as an alternative to the DSM-IV Melancholic Features subtype of Major Depression); ^3^ CORE = CORE Assessment of Psychomotor Change; ^4^ SMPI = Sydney Melancholia Prototype Index; ^5^ Mel = Melancholia.

**Table 2:** Comparison of DSM-IV Major Depression with Melancholia Features, CORE Assessment of Psychomotor Change, and Sydney Melancholia Prototypic Index criteria for identifying melancholia.

DSM-IV	CORE	SMPI
At least one of: 1. Anhedonia 2. Lack of feeling better even when something good happens. Plus at least 3 of: Profound despair; Feeling worse in the morning 5. Waking early Psychomotor agitation/retardation 7. Weight loss 8. Excessive guilt	1. Unresponsive to interviewer 2. Dull/inattentive 3. Immobile face 4. Self-preoccupied 5. Unable to be cheered up 6. Slumped posture 7. Immobility 8. Slowed movements 9. Slowed speech 10. Mute, reduced speech 11. Poverty of associations 12. Impaired insight 13. Nihilistic 14. Observable anxiety 15. Endogenous quality	1. Very low energy 2. Lack of feeling better even when something good happens 3. Feeling worse in the morning 4. Anhedonia 5. Pessimistic 6. Psychomotor retardation 7. Poor concentration 8. Weight loss 9. Profound depression 10. No childhood abuse 11. Usually feels OK when not depressed 12. Depression can come ‘out of the blue’

## Data Availability

No new data were created for this manuscript. All reported data are available in the original cited sources.

## Supplementary Materials

The following supporting information can be downloaded at: https://www.mdpi.com/article/10.3390/neurosci7030074/s1, Supplementary Materials S1: Database search queries; Supplementary Materials S2: PRISMA checklist; Supplementary Materials S3: Quality and risk of bias assessment of melancholia studies.
