# Supplementary material for: EEG Signatures of Melancholia: An Update
Source: NeuroSci. 2026 Jun 21;7(3):74. doi: 10.3390/neurosci7030074 (PMC13306071; doi:10.3390/neurosci7030074)
Supplement: Supplementary file 1 [file neurosci-07-00074-s001.zip › neurosci-4304427-supplementary.pdf]

Supplementary Material S1

Database Search Queries

Pubmed

("Electroencephalography"[Mesh] OR electroencephalograph*[tiab] OR EEG[tiab] OR qEEG[tiab] OR quantitative EEG[tiab] OR "brain wave*"[tiab] OR "resting state"[tiab] OR resting[tiab] OR baseline[tiab] OR "eyes closed"[tiab] OR "eyes open"[tiab] OR "power spectral"[tiab] OR spectral power[tiab] OR band power[tiab] OR "alpha power"[tiab] OR "theta power"[tiab] OR "beta power"[tiab] OR "delta power"[tiab] OR gamma[tiab] OR "peak alpha"[tiab] OR "alpha peak"[tiab] OR "alpha reactivity"[tiab] OR asymmetr*[tiab] OR coherence[tiab] OR "functional connectivity"[tiab] OR connectivity[tiab] OR microstate*[tiab] OR entropy[tiab] OR "long range temporal"[tiab] OR LRTC[tiab] OR aperiodic[tiab] OR "1/f"[tiab] OR "aperiodic exponent"[tiab])
AND
(melanchol*[tiab] OR "melancholic feature*"[tiab] OR "with melancholic features"[tiab]
OR melancholia[tiab] OR "endogenous depression"[tiab] OR endogenomorph*[tiab]
OR "vital depression"[tiab])
AND
(depress*[tiab] OR "major depressive disorder"[tiab] OR MDD[tiab] OR "Depressive Disorder, Major"[Mesh])
NOT
(event-related[tiab] OR ERP[tiab] OR ERPs[tiab] OR "evoked potential*"[tiab] OR task[tiab])
NOT
(sleep[tiab] OR polysomnograph*[tiab])

EMBASE

('electroencephalography'/exp OR eeg:ti,ab OR electroencephalograph*:ti,ab OR qeeg:ti,ab OR 'quantitative eeg':ti,ab OR 'resting state':ti,ab OR resting:ti,ab OR baseline:ti,ab OR 'eyes closed':ti,ab OR 'eyes open':ti,ab OR 'spectral power':ti,ab OR 'band power':ti,ab OR 'alpha power':ti,ab OR 'theta power':ti,ab OR 'beta power':ti,ab OR asymmetr*:ti,ab OR coherence:ti,ab OR connectivity:ti,ab OR 'functional connectivity':ti,ab OR microstate*:ti,ab OR entropy:ti,ab OR lrtc:ti,ab OR aperiodic:ti,ab OR '1/f':ti,ab)
AND
('melancholia'/exp OR melanchol*:ti,ab OR 'melancholic feature*':ti,ab OR 'endogenous depression':ti,ab OR endogenomorph*:ti,ab OR 'vital depression':ti,ab)
AND
('major depression'/exp OR depress*:ti,ab OR 'major depressive disorder':ti,ab OR mdd:ti,ab)
NOT
(erp:ti,ab OR 'event related':ti,ab OR 'evoked potential'/exp OR task:ti,ab OR sleep:ti,ab OR polysomnograph*:ti,ab)

PsycInfo

(DE "Electroencephalography" OR TI(eeg OR electroencephalograph* OR qeeg OR "quantitative eeg" OR "resting state" OR resting OR baseline OR "eyes closed" OR "eyes open" OR "spectral power" OR "band power" OR "alpha power" OR "theta power" OR "beta power" OR asymmetr* OR coherence OR connectivity OR microstate* OR entropy OR lrtc OR aperiodic OR "1/f") OR AB(eeg OR electroencephalograph* OR qeeg OR "quantitative eeg" OR "resting state" OR resting OR baseline OR "eyes closed" OR "eyes open" OR "spectral power" OR "band power" OR "alpha power" OR "theta power" OR "beta power" OR asymmetr* OR coherence OR connectivity OR microstate* OR entropy OR lrtc OR aperiodic OR "1/f"))
AND
(TI(melanchol* OR "melancholic feature*" OR melancholia OR "endogenous depression" OR endogenomorph* OR "vital depression") OR AB(melanchol* OR "melancholic feature*" OR melancholia OR "endogenous depression" OR endogenomorph* OR "vital depression") OR DE "Melancholia")
AND
(DE "Major Depression" OR TI(depress* OR "major depressive disorder" OR mdd)
OR AB(depress* OR "major depressive disorder" OR mdd))
NOT
(TI(erp OR erps OR "event related" OR "evoked potential*" OR task OR sleep OR polysomnograph*) OR AB(erp OR erps OR "event related" OR "evoked potential*" OR task OR sleep OR polysomnograph*))

Scopus

TITLE-ABS-KEY ((eeg OR electroencephalograph* OR qeeg OR "quantitative eeg")
AND
("resting state" OR resting OR baseline OR "eyes closed" OR "eyes open" OR "spectral power" OR "band power" OR "alpha power" OR "theta power" OR "beta power" OR asymmetr* OR coherence OR connectivity OR "functional connectivity" OR microstate* OR entropy OR lrtc OR aperiodic OR "1/f")
AND
(melanchol* OR melancholia OR "melancholic feature*" OR "endogenous depression" OR endogenomorph* OR "vital depression")
AND
(depress* OR "major depressive disorder" OR mdd))
AND NOT
TITLE-ABS-KEY (erp OR erps OR "event related" OR "evoked potential" OR task OR sleep OR polysomnograph*)

Supplementary Material S2

PRISMA Checklist

Section and Topic	Item #	Checklist item	Location where item is reported
**TITLE**			
Title	1	Identify the report as a systematic review.	
**ABSTRACT**			
Abstract	2	See the PRISMA 2020 for Abstracts checklist.	Line 11
**INTRODUCTION**			
Rationale	3	Describe the rationale for the review in the context of existing knowledge.	Line 98
Objectives	4	Provide an explicit statement of the objective(s) or question(s) the review addresses.	Line 98
**METHODS**			
Eligibility criteria	5	Specify the inclusion and exclusion criteria for the review and how studies were grouped for the syntheses.	Line 131
Information sources	6	Specify all databases, registers, websites, organisations, reference lists and other sources searched or consulted to identify studies. Specify the date when each source was last searched or consulted.	Line 112
Search strategy	7	Present the full search strategies for all databases, registers and websites, including any filters and limits used.	Supplementary
Selection process	8	Specify the methods used to decide whether a study met the inclusion criteria of the review, including how many reviewers screened each record and each report retrieved, whether they worked independently, and if applicable, details of automation tools used in the process.	Line 131
Data collection process	9	Specify the methods used to collect data from reports, including how many reviewers collected data from each report, whether they worked independently, any processes for obtaining or confirming data from study investigators, and if applicable, details of automation tools used in the process.	Line 152
Data items	10a	List and define all outcomes for which data were sought. Specify whether all results that were compatible with each outcome domain in each study were sought (e.g. for all measures, time points, analyses), and if not, the methods used to decide which results to collect.	Line 172
	10b	List and define all other variables for which data were sought (e.g. participant and intervention characteristics, funding sources). Describe any assumptions made about any missing or unclear information.	Line 172
Study risk of bias assessment	11	Specify the methods used to assess risk of bias in the included studies, including details of the tool(s) used, how many reviewers assessed each study and whether they worked independently, and if applicable, details of automation tools used in the process.	Line 142
Effect measures	12	Specify for each outcome the effect measure(s) (e.g. risk ratio, mean difference) used in the synthesis or presentation of results.	Line 172
Synthesis methods	13a	Describe the processes used to decide which studies were eligible for each synthesis (e.g. tabulating the study intervention characteristics and comparing against the planned groups for each synthesis (item #5)).	Line 131
	13b	Describe any methods required to prepare the data for presentation or synthesis, such as handling of missing summary statistics, or data conversions.	Line 152
	13c	Describe any methods used to tabulate or visually display results of individual studies and syntheses.	Line 152
	13d	Describe any methods used to synthesize results and provide a rationale for the choice(s). If meta-analysis was performed, describe the model(s), method(s) to identify the presence and extent of statistical heterogeneity, and software package(s) used.	Line 152

Section and Topic	Item #	Checklist item	Location where item is reported
	13e	Describe any methods used to explore possible causes of heterogeneity among study results (e.g. subgroup analysis, meta-regression).	Line 152
	13f	Describe any sensitivity analyses conducted to assess robustness of the synthesized results.	Line 152
Reporting bias assessment	14	Describe any methods used to assess risk of bias due to missing results in a synthesis (arising from reporting biases).	Line 142
Certainty assessment	15	Describe any methods used to assess certainty (or confidence) in the body of evidence for an outcome.	Line 126
**RESULTS**			
Study selection	16a	Describe the results of the search and selection process, from the number of records identified in the search to the number of studies included in the review, ideally using a flow diagram.	Line 165
	16b	Cite studies that might appear to meet the inclusion criteria, but which were excluded, and explain why they were excluded.	Line 128
Study characteristics	17	Cite each included study and present its characteristics.	Line 164
Risk of bias in studies	18	Present assessments of risk of bias for each included study.	Supplementary
Results of individual studies	19	For all outcomes, present, for each study: (a) summary statistics for each group (where appropriate) and (b) an effect estimate and its precision (e.g. confidence/credible interval), ideally using structured tables or plots.	Line 172
Results of syntheses	20a	For each synthesis, briefly summarise the characteristics and risk of bias among contributing studies.	Supplementary
	20b	Present results of all statistical syntheses conducted. If meta-analysis was done, present for each the summary estimate and its precision (e.g. confidence/credible interval) and measures of statistical heterogeneity. If comparing groups, describe the direction of the effect.	Line 172
	20c	Present results of all investigations of possible causes of heterogeneity among study results.	N/A
	20d	Present results of all sensitivity analyses conducted to assess the robustness of the synthesized results.	N/A
Reporting biases	21	Present assessments of risk of bias due to missing results (arising from reporting biases) for each synthesis assessed.	N/A
Certainty of evidence	22	Present assessments of certainty (or confidence) in the body of evidence for each outcome assessed.	Supplementary
**DISCUSSION**			
Discussion	23a	Provide a general interpretation of the results in the context of other evidence.	Line 217
	23b	Discuss any limitations of the evidence included in the review.	Line 398
	23c	Discuss any limitations of the review processes used.	Line 398
	23d	Discuss implications of the results for practice, policy, and future research.	Line 442
**OTHER INFORMATION**			
Registration and protocol	24a	Provide registration information for the review, including register name and registration number, or state that the review was not registered.	Line 122
	24b	Indicate where the review protocol can be accessed, or state that a protocol was not prepared.	Line 122
	24c	Describe and explain any amendments to information provided at registration or in the protocol.	N/A
Support	25	Describe sources of financial or non-financial support for the review, and the role of the funders or sponsors in the review.	Line 465
Competing interests	26	Declare any competing interests of review authors.	Line 469
Availability of data, code and other materials	27	Report which of the following are publicly available and where they can be found: template data collection forms; data extracted from included studies; data used for all analyses; analytic code; any other materials used in the review.	Line 466

Supplementary Material S3

Quality and Risk of Bias Assessment of Melancholia Studies – Reviewer 1

Author	Case Definition	Representativeness	Control Selection	Control Definition	Comparability	Ascertainment of exposure	Method of ascertainment	Non-response rate	Total Stars
Pizzagalli, Nitschke [38]	a)*	a)*	c)	a)*	b)*	b)*	a)*	a)*	7
Pizzagalli, Oakes [39]	a)*	a)*	c)	a)*	b)*	b)*	a)*	a)*	7
Shankman, Sarapas [40]	a)*	a)*	a)*	a)*	b)*	b)* d)	a)*	a)*	8
Quinn, Rennie [41]	a)*	a)*	a)*	a)*	b)*	b)*	a)*	a)*	8
Sharpley, Bitsika [42]	a)*	a)*	a)*	a)*	b)*	d)	a)*	a)*	7
Sharpley, Bitsika [43]	a)*	a)*	a)*	a)*	b)*	d)	a)*	a)*	7
Sharpley, Evans [44]	a)*	a)*	a)*	a)*	b)*	d)	a)*	a)*	7

Quality and Risk of Bias Assessment of Melancholia Studies – Reviewer 2

Author	Case Definition	Representativeness	Control Selection	Control Definition	Comparability	Ascertainment of exposure	Method of ascertainment	Non-response rate	Total Stars
Pizzagalli, Nitschke [38]	a)*	a)*	c)	a)*	b)*	b)*	a)*	a)*	7
Pizzagalli, Oakes [39]	a)*	a)*	c)	a)*	b)*	b)*	a)*	a)*	7
Shankman, Sarapas [40]	a)*	a)*	a)*	a)*	b)*	b)* d)	a)*	a)*	8
Quinn, Rennie [41]	a)*	a)*	a)*	a)*	b)*	b)*	a)*	a)*	8
Sharpley, Bitsika [42]	a)*	a)*	a)*	a)*	b)*	d)	a)*	a)*	7
Sharpley, Bitsika [43]	a)*	a)*	a)*	a)*	b)*	d)	a)*	a)*	7
Sharpley, Evans [44]	a)*	a)*	a)*	a)*	b)*	d)	a)*	a)*	7

Scoring Key: Newcastle-Ottawa Quality Assessment Scale for Case-Control Studies

Note: A study can be awarded a maximum of one star for each numbered item within the Selection and Exposure categories. A maximum of two stars can be given for Comparability.

Selection

1) Is the case definition adequate?
   a) yes, with independent validation *
   b) yes, eg record linkage or based on self-reports
   c) no description

2) Representativeness of the cases
   a) consecutive or obviously representative series of cases *
   b) potential for selection biases or not stated

3) Selection of Controls
   a) community controls *
   b) hospital controls
   c) no description

4) Definition of Controls
   a) no history of disease (endpoint) *
   b) no description of source

Comparability

1) Comparability of cases and controls on the basis of the design or analysis
   a) study controls for ______________ (Select the most important factor.) *
   b) study controls for any additional factor (These criteria could be modified to indicate specific control for a second important factor.) *

Exposure

1) Ascertainment of exposure
   a) secure record (e.g. surgical records) *
   b) structured interview where blind to case/control status *
   c) interview not blinded to case/control status
   d) written self-report or medical record only
   e) no description

2) Same method of ascertainment for cases and controls
   a) yes *
   b) no

3) Non-Response rate
   a) same rate for both groups *
   b) non respondents described
   c) rate different and no designation
